# BUB1B promotes hepatocellular carcinoma progression via activation of the mTORC1 signaling pathway

**DOI:** 10.1002/cam4.3411

**Published:** 2020-09-25

**Authors:** Jiannan Qiu, Shaopeng Zhang, Peng Wang, Hao Wang, Bowen Sha, Hao Peng, Zheng Ju, Jianhua Rao, Ling Lu

**Affiliations:** ^1^ The Affiliated Cancer Hospital ( Jiangsu Cancer Hospital), Nanjing Medical University Nanjing China; ^2^ Hepatobiliary Center of The First Affiliated Hospital, Nanjing Medical University ＆ Research Unit of Liver Transplantation and Transplant Immunology Chinese Academy of Medical Sciences Nanjing China; ^3^ State Key Laboratory of Reproductive Medicine Nanjing Medical University Nanjing China; ^4^ Jiangsu Collaborative Innovation Center of Biomedical Functional Materials College of Chemistry and Materials Science Nanjing Normal University Nanjing China; ^5^ Jiangsu Key Lab of Cancer Biomarkers, Prevention and Treatment Collaborative Innovation Center for Personalized Cancer Medicine Nanjing Medical University Nanjing China

**Keywords:** BUB1B, hepatocellular carcinoma, mTORC1 signaling pathway., prognosis

## Abstract

**Background and Aims:**

Accumulating studies identified that BUB1 mitotic checkpoint serine/threonine kinase B (BUB1B) is integrally involved in the initiation and development of tumors. Nevertheless, the precise biological role and underlying mechanisms of BUB1B in hepatocellular carcinoma (HCC) remain indistinct.

**Method:**

To figure out the role of BUB1B in HCC, we first assessed its expression using The Cancer Genome Atlas (TCGA) and Gene Expression Profiling Interactive Analysis (GEPIA) databases. We then verified BUB1B expression in HCC tissues, nontumor tissues, and HCC cell lines through western blotting, quantitative reverse transcription‐polymerase chain reaction, and immunohistochemistry. To explore the specific function of BUB1B in HCC in vivo and in vitro, we performed the flow cytometry, Cell Counting Kit‐8, 5‐ethynyl‐2′‐deoxyuridine incorporation, colony formation, Transwell, wound‐healing, subcutaneous tumor growth, and metastasis assays. Additionally, we identified the BUB1B‐regulated pathways involved in HCC by using gene set enrichment analysis.

**Results:**

Our data displayed that higher BUB1B expression was detected in HCC tissues and HCC cell lines. The overexpression of BUB1B was positively correlated with adverse clinicopathological characteristics. Survival analyses showed that lower recurrence‐free and overall survival rates were correlated with the overexpression of BUB1B in patients with HCC. Moreover, the malignancy of HCC was facilitated by BUB1B both in vivo and in vitro. Lastly, the results were confirmed by western blots, which showed that BUB1B upregulated mTORC1 signaling pathway in HCC. Meanwhile, the oncogenic effect of BUB1B will be impaired when the mTORC1 signaling pathway was inhibited by rapamycin.

**Conclusion:**

We highlighted that BUB1B played an oncogenic role in HCC and was identified as a possible clinical prognostic factor and a potential novel therapeutic target for HCC.

## Graphical abstract

Our study confirmed that BUB1B is overexpressed in HCC tissues and cell lines, and the overexpression of BUB1B is highly correlated to adverse clinicopathological features, lower recurrence‐free survival and overall survival rate in patients with HCC. Our results revealed the oncogenic role of BUB1B in HCC through a series of functional assays. Moreover, we identified the BUB1B/mTORC1 signaling axis as a critical regulator of the progression in HCC.

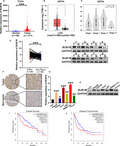

## 1 INTRODUCTION

Hepatocellular carcinoma (HCC) remains one of the most frequent malignant cancers and a principal cause of cancer‐related mortality all over the world. This is especially true for Asia where unfortunately most patients are from China.^1^, ^2^ Hepatitis virus infections, alcoholic liver disease (ALD), intake of *A flavus*, and metabolic‐associated fatty liver disease (MAFLD) are the most common risk factors for HCC.^3^, ^4^, ^5^ With the improvement of modern surgical technology, surgical resection and liver transplantation have become mainstream treatments for HCC.^6^ However, high recurrence and metastases rates result in nonideal postoperative survival.^7^ Hence, there is a sense of urgency to determine the underlying mechanisms of the initiation and development of HCC.^8^

Bub1b (also called BubR1), encoded by BUB1B, belongs to the core elements of the spindle assembly checkpoint (SAC), which includes Mad1, Mad2, Mad3/Bub1b, Mps1, Bub1, and Bub3.^9^ The SAC plays a vital role in arresting cell division until the precise chromosome segregation is ensured during mitosis and meiosis in order to maintain genomic stability.^10^ BUB1B is the mammalian homolog of yeast Mad3, but is markedly distinct from Mad3 on account of a kinase domain possessed by BUB1B.^11^ Growing evidence had demonstrated that aberrant expression of BUB1B was highly involved in the tumorigenesis and development of various tumors. For instance, overexpression of BUB1B is related to chromosomal instability in bladder cancer.^12^ Activation of FoxM1/BUB1B signaling pathway was vital for rhabdomyosarcoma growth and survival.^13^ Moreover, the lung adenocarcinoma progression was dependent on the expression of BUB1B/BUBR1.^14^ Overexpression of BUBR1 was closely correlated with DNA aneuploidy, which may facilitate the progression of gastric cancer.^15^ Further study revealed that BUB1B kinase was frequently overexpressed in high‐grade breast cancer and was prognostic for a worse outcome.^16^ Nevertheless, we still know quite little about the functional roles and potential mechanisms of BUB1B in HCC.

Our present study demonstrates that BUB1B is upregulated in HCC tissues and HCC cell lines compared to adjacent normal liver tissues and normal hepatic cell line, respectively. Survival analyses uncovered that the overexpression of BUB1B owned a positive relation with a shorter recurrence‐free rate and worse overall survival in patients suffering from HCC. BUB1B contributed to the HCC progression by facilitating the proliferation, migration, invasion, epithelial‐mesenchymal transition (EMT), metastases, inhibiting cell apoptosis, and lowering G0/G1 phase arrest of HCC cell lines. Finally, the mTORC1 signaling pathway upregulated by BUB1B was detected, as indicated by gene set enrichment analysis (GSEA). Altogether, our research findings demonstrate that BUB1B is a likely clinical prognostic biomarker and a novel therapeutic target for HCC.

## 2 METHODS AND MATERIALS

### 2.1 Tissue samples and cell lines

We obtained human liver tissue samples consisting of the tumor tissues and adjacent normal liver tissues from 80 patients suffering from HCC who underwent liver partial hepatectomy at the Hepatobiliary Center of the First Affiliated Hospital of Nanjing Medical University. All tissue samples were partly stored in formaldehyde solution and the rest was directly frozen in liquid nitrogen for storage before executing experiments. Our study obtained the consent of the Ethics Committee of the First Affiliated Hospital of Nanjing Medical University before the research began.

The six human HCC cell lines (Hep3B, LM3, SMMC7721, Huh7, HepG2, and Focus) and the normal hepatic cell line L02 were purchased from the American Type Culture Collection (Manassas, VA, USA). We cultured all cells with Dulbecco's modified Eagle's medium (DMEM; Gibco, USA) with the addition of 10% fetal bovine serum (FBS) and 1% antibiotics (streptomycin/penicillin; Gibco, USA) at 37°C in a humidified incubator containing 5% CO_2_.

### 2.2 Lentiviral transfection and treatment

Lentiviral vectors encoding BUB1B (LV‐BUB1B), short‐hairpin RNAs (shRNAs) against BUB1B (LV‐shBUB1B), and corresponding empty vectors (LV‐control/LV‐shNC) were obtained from GenePharma Biotech (Shanghai, China). The infection efficiency was enhanced by adding 5 μg/ml polybrene (Genepharma) to the cell lines.

The stably transfected cell lines were selected by adding 6 μg/ml of puromycin (Beyotime, Guangzhou, China) into the cells for 7 days. In the rescue experiments, we treated the LV‐BUB1B‐transfected HepG2 cells with rapamycin (100 nmol/L).

### 2.3 Cell Counting Kit‐8 assay

The Cell Counting Kit (CCK‐8; Vazyme Biotech Co., Ltd) was utilized to assess cellular proliferative potential on the basis of the manufacturer's protocol. In brief, we planted the stable cell lines into 96‐well plates (500 cells/well) with the addition of CCK‐8 solution (10 μl/well). After incubation for 2 hours in the incubator described as above, we measured the absorbance (450 nm) of each well.

### 2.4 Colony formation assay

We seeded the stable cell lines into 6‐well plates (500 cells/well). After culturing all cells with DMEM for 14 days, we discarded the culture media and washed the cells with PBS two times. Then we fixed the cells with 75% ethyl alcohol for 5 min. Finally, we identified the proliferating colonies by staining them with crystal violet dye solution (Beyotime, Guangzhou, China) after rinsing with PBS three times.

### 2.5 Apoptosis and cell cycle assay

We made use of the Annexin V‐FITC/propidium iodide (PI) Apoptosis Detection Kit (Vazyme Biotech Co., Ltd) to identify apoptotic cells on the basis of the manufacture's protocol. After dissociating the stable cell lines with trypsin without EDTA and centrifuging at 300*g* at 4°C for 5 min, we washed the cells with precooled PBS two times and then pelleted the cells through repeat centrifugation. We collected the cell sediment and resuspended them with 100 μl binding buffer, subsequently added 5 μl FITC‐conjugated Annexin V and 5 μl PI into the cell suspension. At room temperature the reaction lasted for 10 min, during which time it needs to be completely in the dark. At last, the cell suspension was supplemented with 400 μl binding buffer, then the mixture was gently blended. The apoptosis level of cell samples would be detected by the flow cytometry within 1 hour.

For the cell cycle assay, we utilized a Cell Cycle Analysis Kit (Beyotime, Shanghai, China). We first digested the stable cell lines with trypsin for 2 min, then added DMEM to the culture dish to stop the digestion. We collected the cell suspension and centrifuged them at 400 *g* for 5 min. After rinsing the cell lines with PBS and repeating the centrifugation as above, we discarded the PBS and first fixed the cell lines with 4 ml of 75% ethyl alcohol for 30 min, and then stored them at −20°C overnight. The next day, we collected the cell lines after centrifugation at 400 *g* for 5 min, then rinsed them with PBS two times and repeated the centrifugation as above. For cell cycle analysis, it was required that the cells were incubated with 50 mg/ml RNase and 500 μl PI solution for 20 min at room temperature, during which time it needs to be completely in the dark. The data on apoptosis and cell cycle were analyzed by the FlowJo software (Tree Star Inc).

### 2.6 Transwell assays

The Transwell chambers (Millipore, USA) were utilized to evaluate the migratory and invasive capabilities of the cells. The main difference between cell invasion and migration assays is that the Transwell plates (8‐µm pore size) were coated with Matrige for the former and left uncoated for the latter. We planted the cell lines into the upper chamber and cultured them with 200 μl DMEM with no addition of FBS; however, we filled the lower chamber with 500 μl complete medium containing 10% FBS, which attracted the cells across the Transwell membrane into the lower chamber. After one‐day incubation in the incubator described as above, we discarded the culture medium in the upper chamber and washed the Transwell chambers with PBS two times, then fixed the cells with methyl alcohol for 30 min. The invasive or migratory cells were dyed with 0.1% crystal violet for 30 min in the dark at room temperature. The chambers were rinsed with PBS three times and the stained cells were observed through a light microscope.

### 2.7 Wound‐healing assay

We planted the SMMC7721 and HepG2 cell lines (5 × 10^5^ cells/well) into 6‐well plates. After about 48 hours of culture, the cells almost reached confluency. After washing away nonadherent cells two times with PBS, a uniform linear scratch was made using a sterile 200‐μl pipet tip in the center of the well. Taking the distance between the wound edges at 0 hour after rinsing two times with PBS as a baseline, we acquired the image of the same location after 48 hour. The change in the distance was measured to assess the cellular ability of migration and repair.

### 2.8 RNA extraction and qRT‐PCR

We utilized the EasyPure RNA Kit (Ruijie Biotech) to extract total RNA from tissue and cell samples on the basis of the manufacturer's instructions. In short, tissues or cells were mixed with the lysis buffer. After the efficient lysis was confirmed, the lysis solution was collected and vortexed for 10 sec, then added with an equal volume of absolute ethyl alcohol and mixed together. The lysis solution was transferred into an RNA column, which was placed in a centrifuge tube. The tube was centrifuged at 4000*g* for 1 min at 4°C and washed with 500 μl Wash Buffer followed by centrifugation at 12 000*g* for 1 min. Subsequently, the RNA column was transferred into a new and RNase‐free tube and air‐dried for 2 min. We added 20 μl Elution buffer into the center of the RNA column and waited for 2 min. Subsequently, the tube was centrifuged at 12 000 × *g* for 1 min and the RNA extracted stored at −80°C prior to further experiments. For cDNA synthesis a 20 μl/well reaction mix was composed of 1 μg total RNA and 4 μl 5 × HiScript ⅡqRT SuperMix (Vazyme Bioteh co.,ltd) and RNase‐free ddH_2_O.

We performed the qRT‐PCR with the ChamQ^TM^ Universal SYBR qPCR Master Mix (Vazyme Bioteh Co.,Ltd) based on the standard protocols. Primer sequences used in this study for PCR amplification were as follows: BUB1B: 5′‐CTTAGGGTGCAGCTGGATGT‐3′ (forward) and 5′‐ACCCATCCCAGAAGACCTGT‐3′ (reverse); β‐actin: 5′‐TGACGTGGACATCCGCAAAG‐3′ (forward) and 5′‐CTGGAAGGTGGACAGCGAGG‐3′ (reverse). See Supplementary Materials and Methods.

### 2.9 Protein extraction and western blotting

We used the whole protein extraction kit (KeyGEN BioTECH) to extract tissues and cell proteins on the basis of the manufacturer's instructions. All protein samples were separated by 10% SDS‐PAGE and then transferred onto polyvinylidene fluoride (PVDF) membranes. The antibodies used were listed as follows: BUB1B (Abcam), E‐cadherin, N‐cadherin, Vimentin, Bax, cleaved caspase 3, Bcl‐2, CDK2, CDK4, CDK6, mTOR, S6, p‐S6 (at Ser240/244), P70S6K, p‐P70S6K (at Thr389), p‐mTOR (at Ser2448), GAPDH, horseradish peroxidase (HRP)‐conjugated anti‐rabbit IgG antibodies (Cell Signaling Technology). See Supplementary Materials and Methods.

### 2.10 Immunohistochemical (IHC) staining

Once the samples were obtained, we immediately fixed them in 4% formaldehyde solution, then embedded them in the paraffin. We cut the paraffin block into 4‐μm slices and incubated them with primary antibodies for BUB1B and Ki‐67 (Abcam) for 12h at 4°C.

After that, we incubated the slices with secondary antibody conjugated with HRP for 1h at room temperature. Finally, we stained the slices with 3,3′‐diaminobenzidine and hematoxylin for detection. Two independent pathologists in a blind study assessed the positivity and intensity of the staining. Ten fields from each slide were selected randomly for examination.

### 2.11 5‐Ethynyl‐2′‐deoxyuridine (EdU) assay

The EdU assay kit (RiboBio, Guangzhou, China) was used to assess cellular proliferation capacity in the cell lines, on the basis of the manufacturer's instructions. In short, the stable cell lines were planted into 96‐well plates (2 × 10^4^ cells/well) and cultured with the complete medium for 1 day. Subsequently, the cell lines were incubated with 50 μmol/L EdU (RiboBio) for 2 hours at 37°C. Then, we fixed the cell lines with 4% formaldehyde solution for 30 min. Subsequently, the cell lines were permeabilized with 0.5% TritonX‐100 for 10 min. We added 400 μl 1 × ApolloR reaction cocktail and subsequent 400 μl Hoechest33342 to visualize the EdU‐positive cells after rinsing the cell lines with PBS three times. At last, we obtained the images of cells through a microscope.

#### 2.11.1 TUNEL assay

For the purpose of detection of the DNA fragmentation characteristic of apoptosis in formalin‐fixed paraffin‐embedded tumor slices, we performed the TUNEL staining using the Klenow‐FragEL DNA Fragmentation Detection Kit (Roche, Basel, Switzerland) on the basis of the manufacturer's instructions. Ten fields from each slide were selected randomly for examination.

#### 2.11.2 Animal experiments

In this study, all procedures involving animals were full of humanistic care and obtained the consent of the Institutional Animal Care and Use Committee of the Nanjing Medical University before the research began. We purchased 4‐week‐old female BALB/c nude mice from the Department of Laboratory Animal Center of Nanjing Medical University. The stably transfected cell lines (1 × 10^6^ cells) resuspended in 100 μl PBS were injected subcutaneously into the nude mice (n = 6 per group) for subcutaneous tumor growth assays. For metastasis assays, 1 × 10^6^ of HCC cells expressing luciferase were injected into the caudal vein of nude mice (n = 10 per group). After 5 weeks, the occurrence of distant metastases was observed using the IVIS 100 Imaging System (Caliper Life Sciences, Waltham, MA, USA).

#### 2.11.3 Statistical analysis

All data in this study are expressed as mean ± SD. We made use of the SPSS software ver. 20.0 and GraphPad Prism 8.0 to analyze the data and calculate the *p*‐value. Statistical differences between multiple groups were evaluated using Student's t‐test or analysis of variance (ANOVA). All experiments were repeated at least three times and the differences deemed statistically significant when *p*‐value < 0.05.

## 3 RESULTS

### 3.1 BUB1B is overexpressed in HCC and is associated with poor prognosis

**Figure 1:**
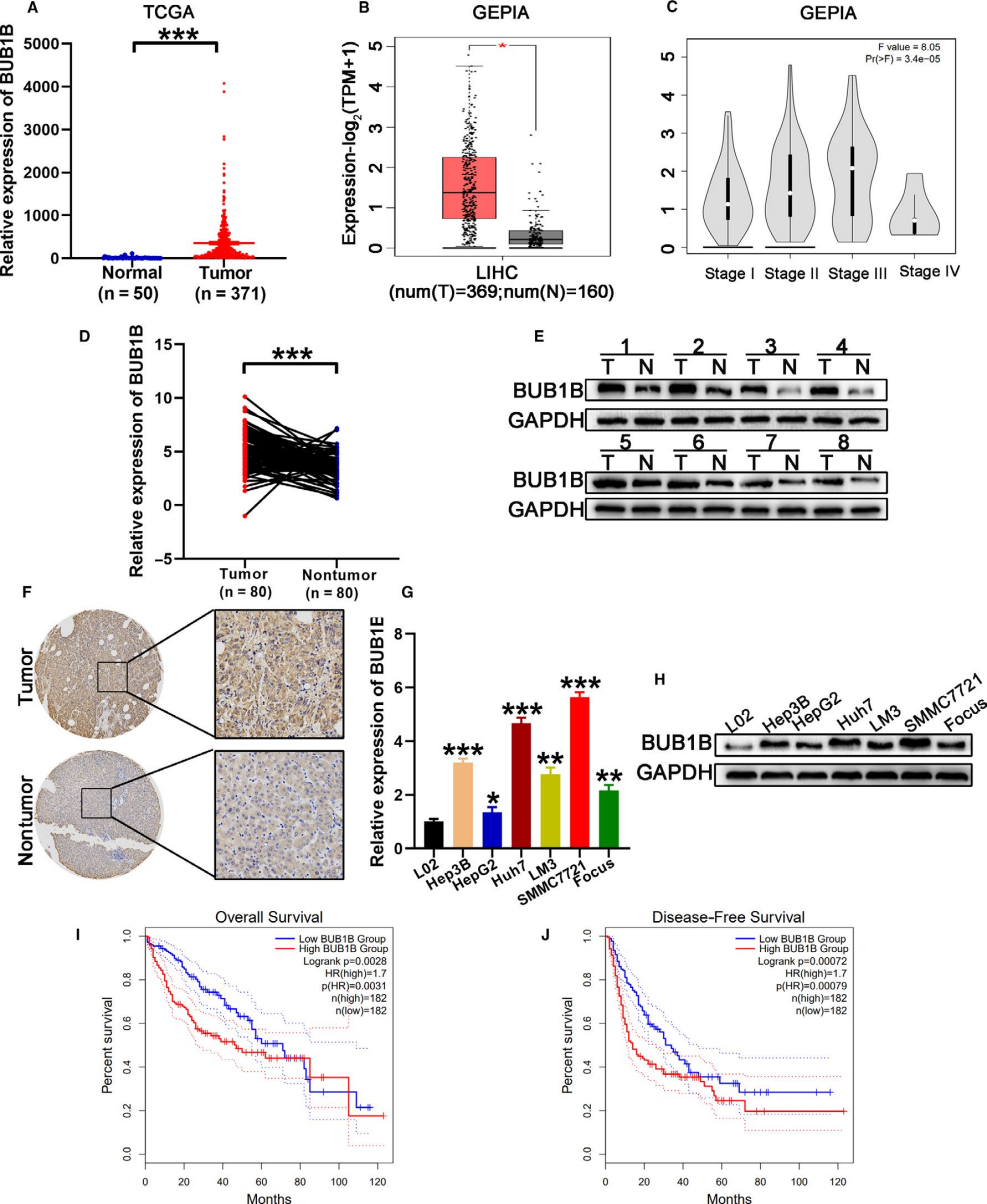
BUB1B is overexpressed in HCC. A, TCGA data analysis of the expression of BUB1B in HCC and normal tissues. B, Expression of BUB1B in HCC and normal tissues analyzed using the GEPIA database. C, Correlation between BUB1B expression and HCC stage was analyzed using the GEPIA database. D, Quantitative RT‐PCR analysis of BUB1B expression in HCC tissues and corresponding normal tissues. E, Western blot analysis of the levels of BUB1B in eight random pairs of HCC tissues (T) and corresponding adjacent normal tissues (N). F, Expression of BUB1B in tumor and normal tissues analyzed by IHC. G,H, Quantitative RT‐PCR and western blot analysis of relative mRNA and protein levels, respectively, of BUB1B in L02 cells and six human HCC cell lines. I,J, Kaplan‐Meier analysis of the relationship of BUB1B with overall survival (i) or recurrence‐free survival (j) in patients with HCC using the GEPIA database. Data represent means ± SD of at least three independent experiments. *P < .05, **P < .01, and ***P < .001

To determine the expression pattern of BUB1B in HCC, we downloaded RNA sequencing data from TCGA (371 HCC tissues and 50 nontumor tissues). The data displayed that the expression of BUB1B was markedly upregulated in HCC tissues than the normal tissues (Figure 1A). In line with TCGA data, the GEPIA database also showed increased BUB1B expression in HCC tissues (Figure 1B). It was revealed that the expression of BUB1B possessed a positive correlation with the HCC stage based on the information obtained from the GEPIA database (Figure 1C). To identify whether BUB1B was markedly overexpressed in HCC tissues, we examined the relative mRNA level of BUB1B in 80 paired HCC tissues and corresponding adjacent nontumor tissues by qRT‐PCR. The results displayed that the relative BUB1B mRNA levels were observably elevated in HCC tissues (Figure 1D). Additionally, the upregulated protein levels of BUB1B were detected, as demonstrated by western blotting and IHC, in eight randomly selected paired HCC tissues (Figure 1E,F). Consistent with these results, we found that the relative BUB1B mRNA and protein levels were elevated in six HCC cell lines including Hep3B, HepG2, Huh7, LM3, SMMC7721, and Focus. In contrast, the normal human hepatic cell line LO2 showed the low BUB1B expression (Figure 1G,H). To investigate whether BUB1B expression was associated with the clinicopathologic features of patients with HCC, we segregated all patients into BUB1B‐high or BUB1B‐low groups. We observed that high levels of BUB1B were significantly associated with larger tumor size, multiple tumors and microvascular invasion, and higher TNM and Edmonson stages (Table 1). Then, the online bioinformatics tool GEPIA database was used for survival analysis and our results showed that HCC patients with higher BUB1B expression were characterized by a shorter recurrence‐free rate and worse overall survival (Figure 1I,J). Taken together, these results demonstrate that BUB1B is overexpressed in HCC in vivo and in vitro, suggesting that BUB1B is probably involved in the tumorigenesis and progression of HCC and a likely prognostic biomarker for HCC.

**Table 1:** Association between BUB1B expression and clinicopathological features in patients with HCC (n = 80)

Characteristics	Number	BUB1B expression		*P*‐value
		Low group	High group	
Age(years)				
<50	23	10	13	.459
≥50	57	30	27	
Gender				
Female	27	10	17	.098
Male	53	30	23	
Cirrhosis				
Present	67	35	32	.363
Absent	13	5	8	
HBV infection				
Positive	70	34	36	.499
Negative	10	6	4	
Tumor size(cm)				
<5	44	31	13	***P* < .001** ***
≥5	36	9	27	
Microvascular invasion				
Presence	38	10	28	***P* < .001** ***
Absence	42	30	12	
Tumor multiplicity				
Simple	38	24	14	**.025** *
Multiple	42	16	26	
α‐Fetoprotein (ng/ml)				
≤20	43	20	23	.501
>20	37	20	17	
TNM stage				
I	36	24	12	**.007** **
II/III	44	16	28	
Edmonson stage				
I/II	42	28	14	**.002** **
III/IV	38	12	26	

* *P* < .05

** *P* < .01

*** *P* < .001

### 3.2 BUB1B contributes to the malignancy of HCC

**Figure 2:**
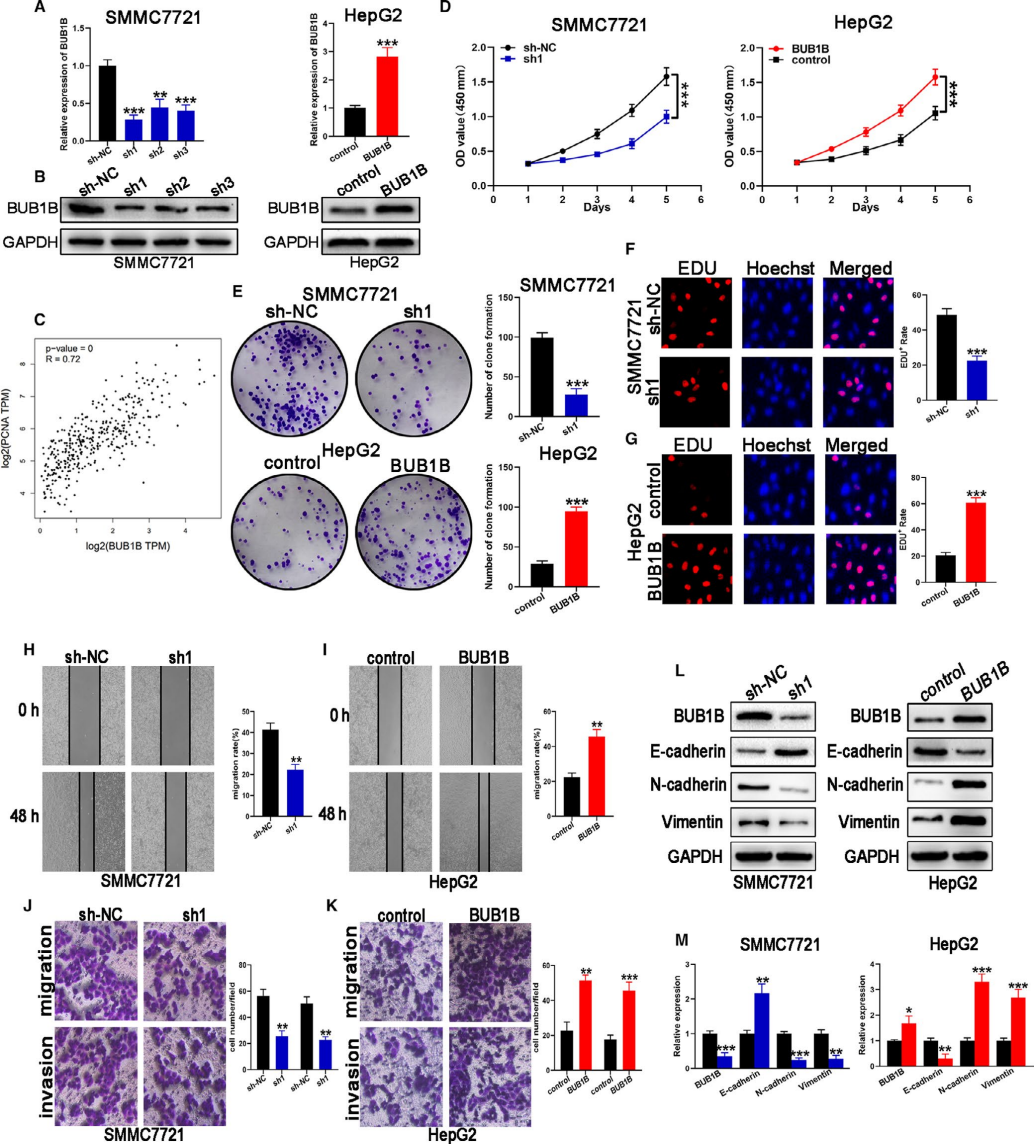
BUB1B contributes to the proliferation, migration, and invasion of HCC cells. A,B, Knockdown and overexpression efficiency of BUB1B were analyzed through qRT‐PCR and western blot, respectively. C, Correlation between BUB1B and PCNA was analyzed using the GEPIA database. D‐G, Cell proliferation in HCC cell lines with BUB1B knockdown or overexpression was assessed with CCK‐8, colony formation, and EDU assays. H, I Wound‐healing assays detected cell migration in cell lines with BUB1B knockdown or overexpression. J,K, Transwell assays of cell migration and invasion of HCC cell lines when BUB1B was knocked down or overexpressed. L,M, Western blot analysis of BUB1B, E‐cadherin, N‐cadherin, and Vimentin expression in HCC cell lines with stably downregulated or overexpressed BUB1B. Data represent means ± SD of at least three independent experiments. *P < .05, **P < .01, and ***P < .001

Based on the western blotting and qRT‐PCR results shown in Figure 1G and 1H, we selected SMMC7721 cells for BUB1B knockdown experiments because it expressed the highest levels of BUB1B among the six HCC cell lines tested. Meanwhile, the low BUB1B‐expressing HepG2 cells were selected for BUB1B overexpression experiments. We used shRNA to downregulate BUB1B expression in SMMC7721 cells. On account of possessing the highest knockdown efficiency, the sh1 was chosen for all subsequent experiments, on basis of the results of qRT‐PCR and western blotting (Figure 2A,B). The low BUB1B‐expressing HepG2 cells were transfected with LV‐BUB1B to upregulate BUB1B expression and the overexpression efficiency was confirmed based on the results shown in Figure 2A,B.

First, the relationship between BUB1B and the proliferating cell nuclear antigen (PCNA) was assessed. We noticed a positive relation between the expression of BUB1B and PCNA (Figure 2C). The CCK‐8 assay results displayed that BUB1B knockdown reduced the proliferative capacity of SMMC7721 cells (Figure 2D). Consistent with this, knockdown of BUB1B contributed to a notable decrease in the clonogenic survival of SMMC7721 cells (Figure 2E). We performed the EdU incorporation assays to assess the effect of BUB1B on actual cell proliferation at multi‐angle in consideration of its specificity and sensibility. The results displayed that BUB1B knockdown inhibited the proliferation of SMMC7721 cells (Figure 2F). Next, we carried out the wound‐healing and transwell assays for the purpose of investigating the effect of BUB1B knockdown on HCC cell migration and invasion. As shown in Figure 2H, the wound‐healing assay results suggested that the migration of SMMC7721 cells was reduced upon BUB1B knocked down. As determined by the Transwell assays, the migratory and invasive capacities of SMMC7721 cells were inhibited once BUB1B was knocked down (Figure 2J). To further elucidate the function of BUB1B, the wound‐healing and Transwell assays were performed using HepG2 cells with high BUB1B expression. As expected, the overexpression of BUB1B markedly augmented the proliferative and migrative capacities of HepG2 cells (Figure 2D,E,G, and I). These results were further confirmed by the Transwell assay, which showed an enhanced migratory and invasive capacity in BUB1B‐overexpressing HepG2 cells (Figure 2K). These data suggested that BUB1B is required for the HCC proliferation, migration, and invasion. It is widely known that EMT is closely involved in the process of migration and invasion of tumor cells. To confirm whether BUB1B promotes cell migration and invasion via EMT, the expression of “EMT master genes” was assessed. Based on the western blotting results, the BUB1B‐downregulated SMMC7721‐sh1 cells were featured with higher expression of E‐cadherin (an epithelial marker) and lower expression of N‐cadherin and Vimentin (mesenchymal markers). The upregulation of BUB1B showed the opposite effect in HepG2 cells (Figure 2L,M). These data demonstrate that BUB1B modulates cellular migration and invasion by promoting EMT in HCC cell lines.

### 3.3 Overexpression of BUB1B inhibits apoptosis and prevents G0/G1 cell cycle arrest in HCC cells

**Figure 3:**
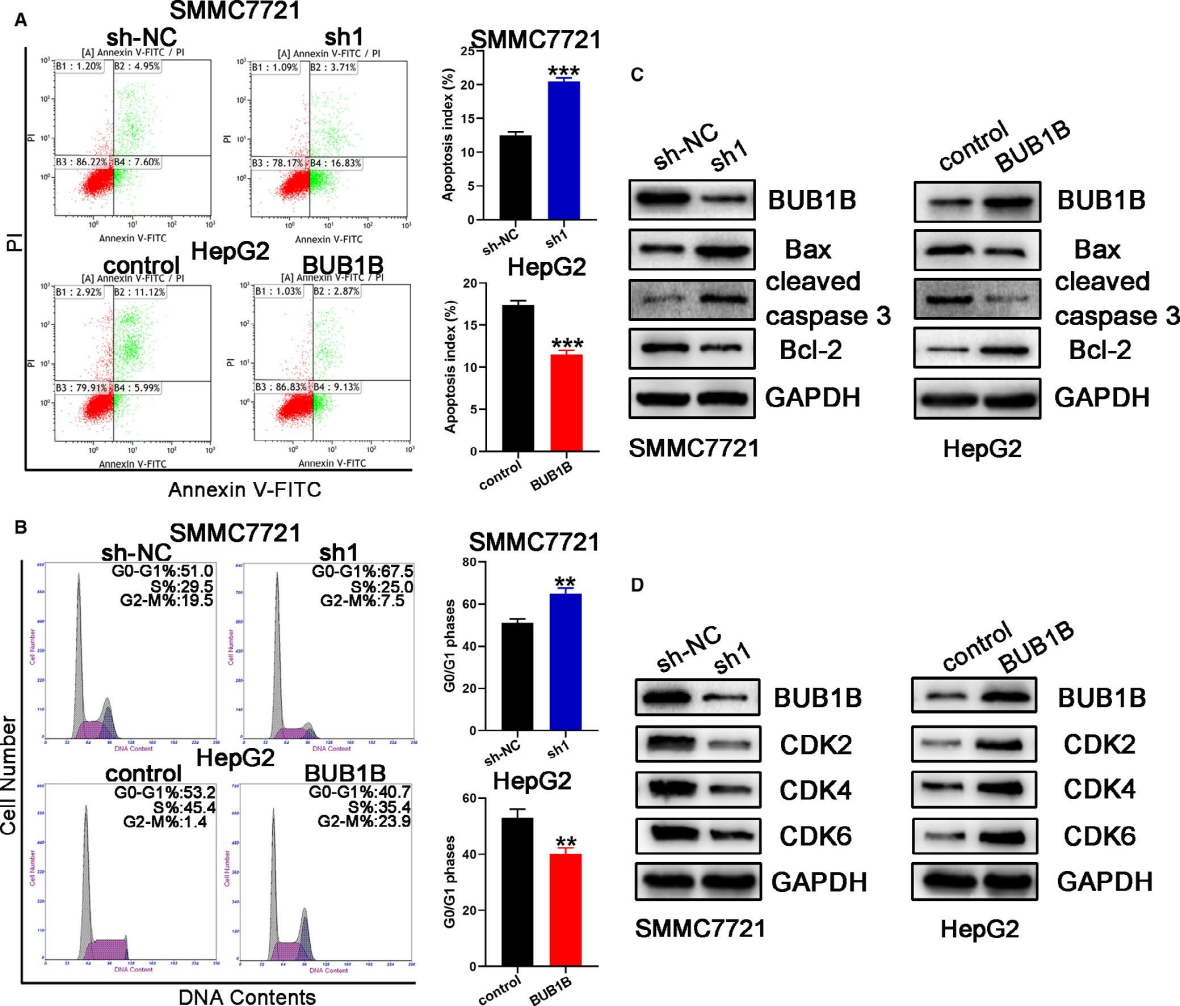
Overexpression of BUB1B inhibited apoptosis and reduced G0/G1 cell cycle arrest in HCC cells. A, Apoptosis of HCC cell lines with BUB1B knockdown or overexpression analyzed by flow cytometry. B, Western blot analysis of apoptosis‐related proteins in HCC cell lines with BUB1B knockdown or overexpression. C, Cell cycle in HCC cell lines with BUB1B knockdown or overexpression analyzed by flow cytometry. D, Western blot analysis of the levels of checkpoint proteins in the G1/S phase. Data represent means ± SD of at least three independent experiments. *P < .05, **P < .01, and ***P < .001

Rate of apoptosis and cell cycle alteration are two important factors in the progression and development of HCC. We performed the flow cytometry experiments to investigate the rate of apoptosis and the cell cycle profile. Our results displayed that the knockdown of BUB1B promoted the apoptosis of SMMC7721 cells, while overexpression of BUB1B in HepG2 cells reduced cellular apoptosis (Figure 3A). Moreover, based on the western blotting results, the protein levels of the apoptosis‐related protein cleaved caspase 3 and Bax were elevated, but the protein levels of the antiapoptotic protein Bcl‐2 were reduced in SMMC7721 cells upon BUB1B knocked down. However, the contrary effects were observed in BUB1B‐overexpressing HepG2 cells (Figure 3A,C).

We then investigated whether BUB1B influenced the cell cycle of HCC cells. The results displayed that the downregulation of BUB1B increased the percentage of G0/G1 phase in the SMMC7721 cells, as demonstrated by the cell cycle assay (Figure 3B). We observed the opposite results in BUB1B‐overexpressing HepG2 cells: lower percentage of G0/G1 phase in the HepG2 cells (Figure 3B). Consistent with this, the protein levels of the G1/S phase checkpoint proteins (CDK2, CDK4, and CDK6) were significantly reduced in BUB1B‐downregulated SMMC7721‐sh1 cells, while the contrary results were obtained in BUB1B‐overexpressing HepG2 cells (Figure 3D).

### 3.4 *BUB1B contributes to xenograft tumor growth and HCC metastasis* in vivo

**Figure 4:**
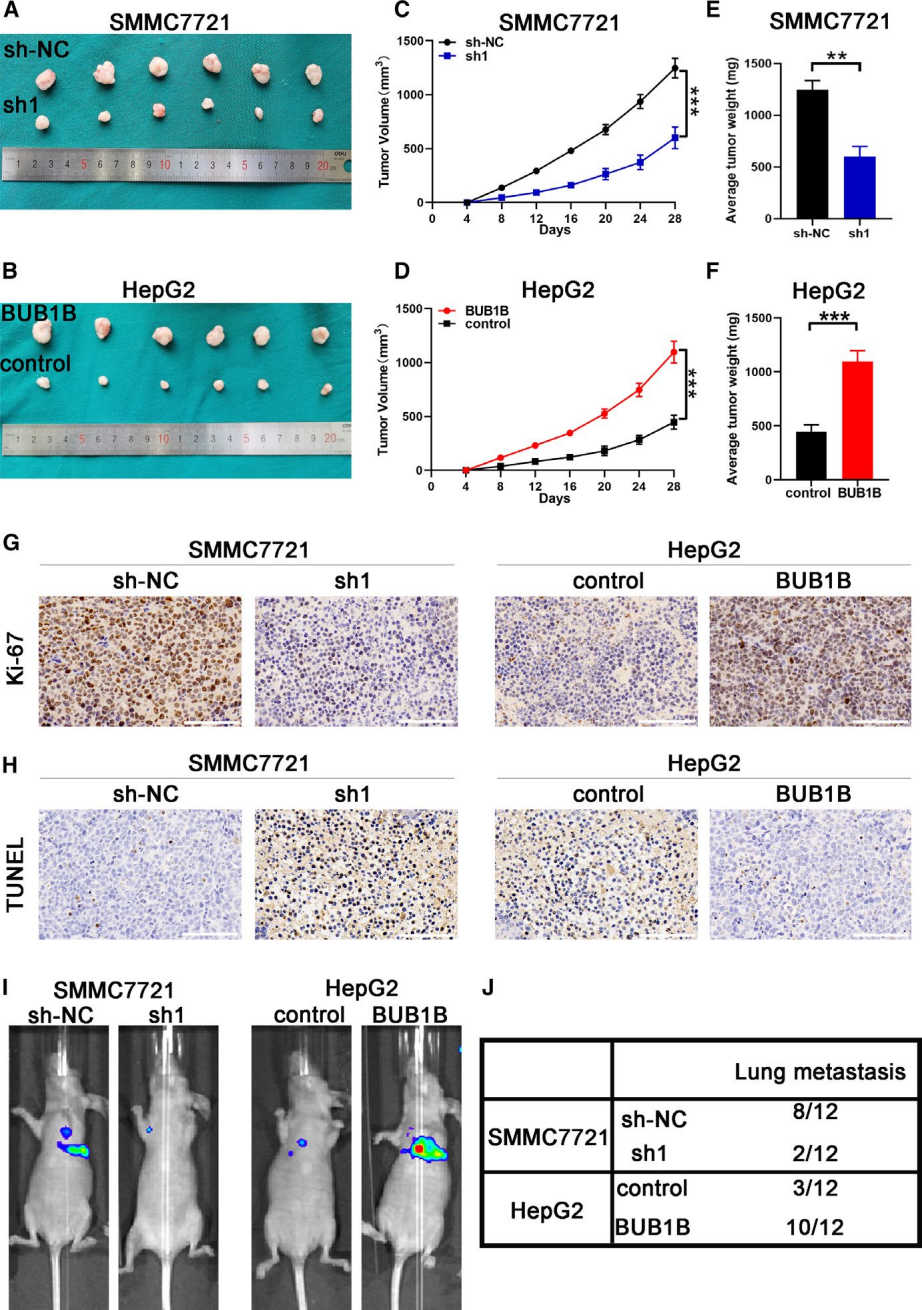
BUB1B contributes to HCC growth and metastasis in vivo. A,B, Representative images of xenograft HCC tumors obtained from mice inoculated with SMMC7721‐sh1 and SMMC7721‐shNC or HepG2‐BUB1B and HepG2‐control. C,D, Growth curves for the tumor volumes were calculated for each group. E,F, Tumor weight of xenograft HCC tumors in each group. G,H, Representative images from IHC for Ki‐67, and TUNEL expression in xenograft HCC tumors from each group. Scale bar, 100 μm. I, Representative bioluminescence imaging of the caudal vein injection mouse model. J, The number of mice with pulmonary metastases is shown in the table. Data represent means ± SD of at least three independent experiments. *P < .05, **P < .01, and ***P < .001

We generated subcutaneous xenograft tumor mouse models of HCC to further examine the effect of BUB1B on tumor progression. Tumors from mice injected with SMMC7721‐sh1 cells showed the slower growth and lower average tumor volume and weight (Figure 4A,C, and E). Conversely, tumors from mice injected with BUB1B‐overexpressing HepG2 cells were characterized by the faster growth and higher average tumor volume and weight (Figure 4B,D, and F). The above data indicated that BUB1B can facilitate tumor progression in vivo. We performed the Ki‐67 staining and TUNEL assays on the tumors to confirm that BUB1B promoted tumorigenicity. Our results demonstrated that the SMMC7721‐sh1 xenografts possessed the lower proliferative activity and the enhanced apoptosis compared to the SMMC7721‐shNC xenografts. In contrast, the upregulation of BUB1B resulted in the increased proliferative activity and reduced apoptosis in HepG2 xenografts (Figure 4G,H). To further investigate whether BUB1B affects HCC metastases in vivo, we injected the luciferase‐expressing and stable‐transfected HCC cells into BALB/c nude mice through the caudal vein. The downregulation of BUB1B in SMMC7721 cells resulted in the fewer lung metastases compared to the corresponding control group, whereas an inverse trend was observed in the HepG2‐BUB1B‐overexpressing group (Figure 4I). We counted the number of mice with pulmonary metastases in each group, as shown in Figure 4J. Taken together, our data suggest that BUB1B can promote tumor progression and metastasis in vivo.

### 3.5 BUB1B activates the mTORC1 signaling pathway

**Figure 5:**
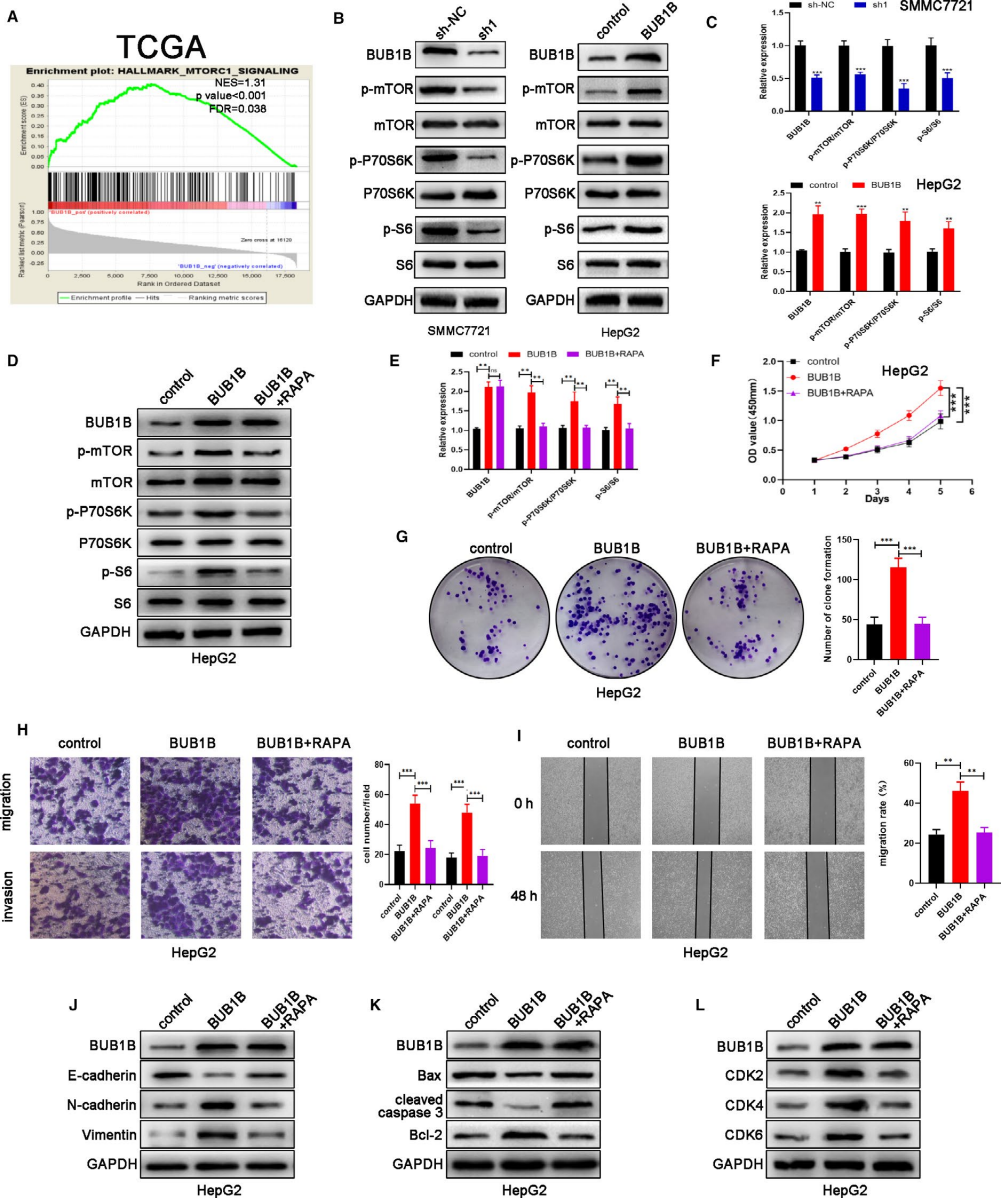
BUB1B activates the mTORC1 signaling pathway. A, The signaling pathway affected by BUB1B analyzed by GSEA based on TCGA data. B,C, Western blot analysis of the levels of several core components involved in the mTORC1 signaling pathway. D,E, Western blot analysis of the levels of several core components involved in the mTORC1 signaling pathway when HepG2 was treated with rapamycin (RAPA). F,G, Cell proliferation in BUB1B‐overexpressing HepG2 cells with the addition of RAPA was assessed with CCK‐8 and colony formation assays. H, Transwell assays of cell migration and invasion of BUB1B‐overexpressing HepG2 cells with addition of RAPA. I, Wound‐healing assays detected cell migration in HepG2 cells from each group. J, Western blot analysis of BUB1B, E‐cadherin, N‐cadherin, and Vimentin expression in HepG2 cells from each group. K, Western blot analysis of apoptosis‐related proteins in HepG2 cells from each group. L, Western blot analysis of the levels of checkpoint proteins in the G1/S phase in HepG2 cells from each group. Data represent means ± SD of at least three independent experiments. *P < .05, **P < .01, and ***P < .001

To further determine the potential signaling pathways modulated by BUB1B, we performed GSEA via analyzing the data from TCGA. We observed that the activity of the mTORC1 signaling pathway was positively correlated with BUB1B expression levels in 371 HCC tissues (Figure 5A). We detected several core proteins involved in the mTORC1 signaling pathway through western blotting. The protein levels of p‐mTOR, p‐P70S6K, and p‐S6 were significantly decreased in BUB1B‐downregulated SMMC7721 cells. In contrast, the protein levels of the core protein described as above were elevated in BUB1B‐overexpressing HepG2 cells (Figure 5B,C). Hence, our data suggest that BUB1B regulates the mTORC1 signaling pathway. Then, we treated BUB1B‐overexpressing HepG2 cells with mTORC1 inhibitor rapamycin (RAPA, 100 nmol/L) for 48 hours; the western blotting results showed the upregulated protein in the mTORC1 signaling pathway was significantly inhibited (Figure 5D,E). The CCK‐8 assays results displayed that rapamycin reduced the elevated proliferative capacity of BUB1B‐overexpressing HepG2 cells (Figure 5F). Consistent with this, rapamycin contributed to a notable decrease in the clonogenic survival of BUB1B‐overexpressing HepG2 cells (Figure 5G). As shown in Figure 5H, the Transwell assays results suggested that the migratory and invasive capacities of BUB1B‐overexpressing HepG2 cells were inhibited by rapamycin. Meanwhile, the wound‐healing assay results showed rapamycin reduced the migration of BUB1B‐overexpressing HepG2 cells (Figure 5I). Based on the western blotting results, rapamycin increased the expression of E‐cadherin and reduced the expression of N‐cadherin and Vimentin (Figure 5J). As shown in Figure 5K and L, the rapamycin‐treated BUB1B‐overexpressing HepG2 cells were featured with the higher expression of Bax and cleaved caspase 3 and the lower expression of Bcl‐2, CDK2, CDK4, and CDK6. All above data suggest that BUB1B plays the oncogenic effect in HCC via activating the mTORC1 signaling pathway.

## 4 DISCUSSION

Recent studies have shown that BUB1B has a variety of effects on the initiation and progression of multiple tumors. It has been reported that the loss of acetylation of the Bub1b results in defects in spindle assembly checkpoint signaling and promotes tumor formation.^17^ Germline mutations in BUB1B were at high risk for the development of early onset colorectal cancer.^18^ Overexpression of BUB1B was significantly correlated with worse clinicopathological characteristics, which predicted tumor recurrence and progression in bladder cancer.^12^ Activation of Forkhead box protein M1/BUB1B signaling promoted the tumorigenesis and radioresistance of glioblastoma through a direct binding between Forkhead box protein M1 and BUB1B.^19^ It has been reported that a germline homozygous intrinsic mutation in the gene BUB1B resulted in an increased susceptibility to gastrointestinal oncogenesis.^20^ Interestingly, it was reported that low expression of BUB1B resulted in the initiation and progression of human colon adenocarcinomas.^21^ The tumorigenesis and metastasis of HCC is a complicated and multi‐faceted process, which is affected by many factors, including epigenetic and genetic factors.^22^ To date, studies that focus on understanding their function in HCC are less clear. Therefore, the specific roles and the definite molecular mechanisms of BUB1B in the initiation and progression of HCC need to be elucidated. In our present study, we evaluated the expression of BUB1B in HCC using TCGA, GEPIA, IHC, qRT‐PCR, and western blotting, and subsequently validated its overexpression in the tumor tissues and adjacent normal tissues from patients suffering from HCC and HCC cell lines. By collecting and analyzing clinical data, we discovered that the expression of BUB1B was closely correlated with worse clinicopathologic features, including larger tumor size, multiple tumors, microvascular invasion, and higher TNM and Edmonson stages. Moreover, HCC patients with higher BUB1B expression had shorter recurrence‐free survival (hazard ratio = 1.7; *P* = .00072) and worse overall survival (hazard ratio = 1.7; *P* = .0028). Given the clinicopathologic significance of BUB1B expression that is associated with HCC progression and poorly prognosis, we evaluated the function of BUB1B in the malignancy of HCC cell lines both in vivo and in vitro. Functional analysis demonstrated that the upregulation of BUB1B contributed to the proliferation, migration, invasion, and metastases of HCC cells. It is widely known that EMT is highly involved in HCC progression and metastases.^23^ As demonstrated by the western blotting results, we found that the upregulation of BUB1B facilitate EMT in HepG2 cells. The facilitated EMT in the HepG2‐BUB1B‐overexpressing group may account for a larger number of distant lung metastases. Apoptosis and the cell cycle play a significant role in regulating the tumorigenesis and progression of HCC.^24^, ^25^ Our results verified that the upregulation of BUB1B inhibited the rate of apoptosis and G0/G1 arrest of HCC cells, indicating that BUB1B can promote the malignant behavior of HCC cells by modulating apoptosis and the cell cycle checkpoints.

BUB1B was identified as an oncogene in prostate cancer through BUB1B‐dependent regulation of MELK transcription.^26^ In multiple myeloma (MM), BUB1B expression is closely associated with cell‐division cycle protein 20 and cyclinB1/2 expression, resulting in the increased proliferation of MM cells, accounting for the oncogenic role of BUB1B.^27^ By using GSEA analysis, we found that mTORC1 signaling pathway was strongly correlated with higher BUB1B expression levels in HCC, indicating that mTORC1 signaling pathway acts the downstream of BUB1B in HCC.

It is well known that the serine/threonine kinase mammalian target of rapamycin (mTOR) is a pivotal kinase that governs cell proliferation, growth, and survival under diverse environmental stimuli, including oxygen, amino acids, energy, growth factors, and stress.^28^, ^29^ Mammalian TOR forms the catalytic subunit of two diverse protein complexes, consisting of mTOR complex 1 (mTORC1) and mTOR complex 2 (mTORC2).^30^ Accumulative studies had reported that the mTORC1 signaling pathway is involved in plenty of physiological processes and its dysregulation is associated with multiple pathologies, ranging from metabolic disorders to tumorigenesis.^30^, ^31^, ^32^ The dysregulation of the mTORC1 signaling pathway has been widely accepted to influence malignant processes by affecting cell proliferation, apoptosis, stemness, drug resistance, and metabolic reprogramming. The mTOR‐S6K pathway inhibited the E3 ligase activity of RNF168 through the phosphorylation of RNF168 at Ser60, resulting in the cumulative unrepaired DNA and genome instability, which account for the initiation of tumors.^33^ The upregulation of BNIP3 enhanced the activation of autophagy and anoikis resistance of HCC cells by suppressing mTOR/S6K1 signaling pathway.^34^ Tripartite motif‐containing 7 suppressed HCC progression through modulation of the Src‐mTORC1‐S6K1 axis.^35^ In line with the above findings, our data displayed that BUB1B could stimulate the mTORC1 signaling pathway to promote HCC progression, which was a brand‐new mechanism of BUB1B in the progression of tumor. Furthermore, the oncogenic effect of BUB1B was inhibited when BUB1B‐overexpressing HepG2 cells were treated with rapamycin (an mTORC1 inhibitor). Based on our data, the proliferation, colony formation, migration, and invasion of BUB1B‐overexpressing HepG2 cells were inhibited by rapamycin. Meanwhile, compared to the BUB1B‐overexpressing HepG2 cells, the rapamycin‐treated BUB1B‐overexpressing HepG2 cells were featured with a lower expression of N‐cadherin and Vimentin but a higher expression of E‐cadherin, which suggested the ability of EMT was suppressed. Also, the rapamycin‐treated BUB1B‐overexpressing HepG2 cells showed a higher expression of Bax and cleaved caspase 3 and a lower expression of Bcl‐2, which suggested the reduced apoptosis of BUB1B‐overexpressing HepG2 cells was abolished when the mTORC1 signaling pathway was inhibited by rapamycin. Next, the protein levels of the G1/S phase checkpoint proteins (CDK2, CDK4, and CDK6) were significantly reduced in rapamycin‐treated BUB1B‐overexpressing HepG2 cells. To sum up, our data indicate that BUB1B promotes HCC progression via activation of the mTORC1 signaling pathway. When the mTORC1 signaling pathway is inhibited, the oncogenic effect of BUB1B will be impaired. As has been reported in previous studies, the aberrant activation of mTORC1 signaling pathway was closely involved in the progression of HCC. For example, the loss of ventricular zone expressed pleckstrin homology domain‐containing 1 (VEPH1) will activate the mTORC1 signaling pathway to promote HCC progression, and rapamycin can serve as an effective therapeutic drug.^36^ Inhibition of mTORC1 enhanced antitumor capability of ubiquitin‐specific protease 10 by stabilizing phosphatase and tensin homolog (PTEN) and 5′ AMP‐activated protein kinase α (AMPKα) in HCC cells.^32^ Melatonin enhanced the Hep3B cells sensitivity to sorafenib by reducing the HIF‐1α protein level through inhibiting the mTORC1/p70S6K/S6 pathway.^37^ Hence, the inhibition of mTORC1 signaling pathway could be the potential therapy to HCC. To our best knowledge, this is the first basic research that reveals a definite oncogenic role of BUB1B in HCC and the relevant mechanism. Nonetheless, additional experiments need to be performed to further investigate the detailed mechanism of BUB1B in HCC tumorigenesis and progression.

In summary, our study confirms that BUB1B is overexpressed in HCC tissues and cell lines. The overexpression of BUB1B is highly correlated with adverse clinicopathological features, lower recurrence‐free survival, and overall survival rate in patients suffering from HCC. Our results revealed the oncogenic role of BUB1B in HCC through a series of functional assays. Moreover, we identified the BUB1B/mTORC1 signaling axis as a critical regulator of the progression in HCC. Future research should focus on the BUB1B/mTORC1 signaling axis, which can serve as a prognostic biomarker and possibly offer a novel therapeutic strategy for the treatment of HCC.

## CONFLICT OF INTEREST

All authors of this study confirm that they have absolutely no conflict of interest concerning this manuscript.

## AUTHOR CONTRIBUTIONS

LL, JR, and JQ conceived of the project and designed the research. JQ, SZ, PW, HW, BS, HP, and ZJ carried out experiments in vivo and vitro. JQ accomplished the manuscript. SZ and PW performed the data analysis. LL and JR offered a lot of suggestions. LL and JQ revised the manuscript. All the authors read and approved the final version to be published.

## Supporting information

Supplementary Materials and Methods

## Data Availability

All data that obtained and analyzed during our study are available from the corresponding author once reasonably requested.
