# Supplementary material for: BUB1B promotes hepatocellular carcinoma progression via activation of the mTORC1 signaling pathway
Source: Cancer Med. 2020 Sep 25;9(21):8159–72. doi: 10.1002/cam4.3411 (PMC7643650; doi:10.1002/cam4.3411)
Supplement: Supplementary file 1 — Supplementary Materials and Methods [file CAM4-9-8159-s001.docx]

**Supplementary materials and methods:**

***1.RNA extraction and quantitative reverse transcription-polymerase chain reaction*** ***(qRT-PCR)***

The EasyPure RNA Kit (Ruijie Biotech) was used to extract total RNA from tissue and cell samples according to the manufacturer’s instructions. To extract tissue RNA, we cut tissues into pieces with a scissor. Tissue pieces per 5 mg were put into the centrifuge tube with 300 μl Lysis Buffer and transferred into a tissue homogenizer for 2 min. For efficient lysis, the tube was placed on ice for 5 min after vortexing. Next, the tube was centrifuged at 12,000 × *g* for 2 min at 4 °C. The supernatant was transferred to a precooled tube. To extract RNA from cells, we discarded the culture media and washed the cells with PBS. Then 500 μl Lysis buffer was added to the culture dish. After lysis, the cell solution was collected and vortexed for 10 sec. An equal volume of absolute ethyl alcohol was added and mixed. The lysis solution was transferred into an RNA column, which was placed in a centrifuge tube. The tube was centrifuged at 4,000 × *g* for 1 min at 4 °C and washed with 500 μl Wash Buffer followed by centrifugation at 12,000 × *g* for 1 min. Subsequently, the RNA column was transferred into a new and RNase-free tube and air-dried for 2 min. We added 20 μl Elution buffer into the center of the RNA column and waited for 2 min. Subsequently, the tube was centrifuged at 12,000 × *g* for 1 min and the RNA extracted stored at -80 °C prior to further experiments.

For cDNA synthesis a 20 μl/well reaction mix was set up with 1 μg total RNA and 4 μl 5×HiScript ⅡqRT SuperMix (Vazyme Bioteh co.,ltd) and RNase-free ddH_2_O. The qRT-PCR was performed by using the ChamQ^TM^ Universal SYBR qPCR Master Mix (Vazyme Bioteh Co.,Ltd) based on standard protocols. Primer sequences used in this study for PCR amplification were as follows: BUB1B: 5′-CTTAGGGTGCAGCTGGATGT-3′ (forward) and 5′-ACCCATCCCAGAAGACCTGT-3′ (reverse); β-actin: 5′-TGACGTGGACATCCGCAAAG-3′ (forward) and 5′-CTGGAAGGTGGACAGCGAGG-3′ (reverse).

***2.9.*** ***Protein extraction and western blotting***

The whole protein extraction kit (KeyGEN BioTECH) was used to extract proteins from cells and tissues, according to the manufacturer's instructions. We made per 1 ml lysis solution with 1 ml Lysis Buffer with 10 μl phosphatase inhibitors, 1 μl protease inhibitors and 10 μl of 100 mM phenylmethylsulfonyl fluoride. The tube containing the lysis solution was placed on ice to maintain a low temperature. To extract tissue proteins, the tissues were cut into pieces with a scissor. The pieces were transferred to the tube with the lysis solution and homogenized for 2 min. Then the tissue homogenate was transferred to a precooled tube and centrifuged at 12,000 × *g* for 5 min at 4 °C. Subsequently, the supernatant, containing the whole protein extract, was transferred to a precooled tube. To extract protein from the cells, the culture medium was removed and the cells were washed with precooled PBS twice. The lysis solution was added to the culture dish and then collected the cells with a cell scraper. The cell suspension was transferred to a precooled tube and placed on ice for 30 min to ensure sufficient lysis. During lysis, the tube was vigorously shaken on a shaking table platform for 30 s every five min. Then the tube was centrifuged at 12,000 × *g* for 5 min at 4 °C and the supernatant, containing the whole protein extract, was transferred to a precooled tube. The protein extracts were mixed with sodium dodecyl sulfate-polyacrylamide gel electrophoresis (SDS-PAGE) loading buffer in a centrifuge tube, which was then placed in boiling water for 10 min. Finally, the protein was stored at -80 °C and repeated freeze-thaw was avoided.

Protein samples were separated by 10% SDS-PAGE and transferred onto polyvinylidene fluoride (PVDF) membranes. After blocking the PVDF membranes in QuickBlock™ Blocking Buffer (Beyotime Biotechnology) for at least 30 min, we incubated them with specific primary antibodies at 4 °C for 12 h. After washing the PVDF membranes with Tris-buffered saline (TBST) buffer for 30 min, we incubated them with horseradish peroxidase (HRP) -conjugated anti-rabbit IgG antibodies at room temperature for 2 h. Finally, the proteins were detected using the Super ECL Detection Reagent (Yeasen Biotech Co., Ltd) after rinsing with TBST buffer for 30 min. The antibodies used were as follows: BUB1B (Abcam), E-cadherin, N-cadherin, Vimentin, Bax, cleaved caspase 3, Bcl-2, CDK2, CDK4, CDK6, mTOR, p-mTOR (at Ser2448), P70S6K, p-P70S6K (at Thr389), S6, p-S6 (at Ser240/244), GAPDH (Cell Signaling Technology).
